# Is AI the Future of Mental Healthcare?

**DOI:** 10.1007/s11245-023-09932-3

**Published:** 2023-05-31

**Authors:** Francesca Minerva, Alberto Giubilini

**Affiliations:** 1 Department of Philosophy, University of Milan, Milano, Italy; 2 Oxford Uehiro Centre for Practical Ethics, University of Oxford, Oxford, England

Over the past decade, AI has been used to aid or even replace humans in many professional fields. There are now robots delivering groceries or working in assembling lines in factories, and there are AI assistants scheduling meetings or answering the phone line of customer services. Perhaps even more surprisingly, we have recently started admiring visual art produced by AI, and reading essays and poetry “written” by AI (Miller 2019), that is, composed by imitating or assembling human compositions. Very recently, the development of ChatGPT has shown how AI could have applications in education (Kung et al. 2023) the judicial system (Parikh et al. 2019) and the entertainment industry.^1^

One of the most promising areas of development for AI is healthcare (Mishra et al. 2021). AI has been used for a few years to assist in diagnosing patients and find the best treatments (see, for instance, IBM Watson). More recently, AI robots have been used to help surgeons perform brain surgery (Prabu et al. 2014).

AI is quickly becoming effective at performing several tasks in healthcare settings that we used to consider a human prerogative. In particular, AI seems to be better than humans at diagnosing some diseases because it can learn from vast datasets and recognise patterns better than we can (Loh 2018). It is therefore likely that areas of medicine mainly concerned with diagnosing will be integrated with AI sooner than others.

Demand for healthcare assistance is constantly increasing, but financial resources are limited. The prospect of using AI to provide substantial support with the administration of healthcare has made many hopeful about the possibility of bettering healthcare assistance worldwide and making it more cost-effective. Reports of its successful use in medicine have appeared in both scientific journals and popular magazines. For instance, some studies suggest that AI is better than human doctors at detecting conditions such as skin cancers (Esteva and Topol 2019) or diabetic retinopathy (Savoy 2020), and some predict that AI will replace human healthcare practitioners within a few decades^2^.

Assuming that AI can deliver on what it promises—a big assumption we take for granted in this article—AI could allow better care for a greater number of people. Yet the costs of switching from human to AI medical assistance cannot be dismissed.

One of the obvious costs associated with replacing a significant number of human doctors with AI is the dehumanization of healthcare. The human dimension of the therapist-patient relationship would surely be diminished. With it, features of human interactions that are typically considered a core aspect of healthcare provision, such as empathy and trust, risk being lost as well. Sometimes, the risk of dehumanizing healthcare by having machines instead of persons dealing with patients might be worth taking, for instance when the expected outcomes for the patient are significantly better. However, some areas of healthcare seem to require a human component that cannot be delegated to artificial intelligence. In particular, it seems unlikely that AI will ever be able to empathize with a patient, relate to their emotional state or provide the patient with the kind of connection that a human doctor can provide. Quite obviously, empathy is an eminently human dimension that it would be difficult, or perhaps conceptually impossible, to encode in an algorithm.

In some areas of healthcare, these factors might be almost irrelevant. One might care little about having a human connection with the entity (human or not) filling a cavity or performing surgery on a broken finger. But in areas such as psychiatry and mental health care, interaction with another human is likely to be irreplaceable, as it seems to be one of the main factors contributing to successful psychiatric diagnosis and treatment.

However, if using AI in psychiatry yields positive results for the patients, not only would the dehumanisation of psychiatry be a cost worth paying—especially if it reduces costs and improves access; in addition, the very nature of psychiatry as essentially grounded in the human connection between the therapist and the patient would be called into question. Even if there are reasons to be skeptical about the possibility of this radical change of perspective, it is worth looking at what the potential benefits might be. It is possible that further down the line, we will be surprised by what the use of AI in psychiatry can achieve, just as 20 or 30 years ago we would have been surprised if someone had claimed that smartphones were going to become such a big part of our lives, or that AI was going to become so prominent in academic discussion. And yet, we spend several hours a day on our smartphones and the potential benefits and downside of AI have become one of the most debated issues both inside and outside of academia.

## Mental Healthcare

When thinking of mental healthcare, most of us probably picture a patient lying down on a sofa, talking about their latest nightmare, or their bad relationship with their mother, to a therapist who takes notes in a journal. Indeed, this has been the paradigm in mental health care for a very long time. Psychoanalysis has been popular since it was first introduced by Sigmund Freud in the late 19th century. Behavioural psychotherapy has gained popularity since the ‘60s of the last Century. There are certainly relevant differences between psychoanalysis and behavioural psychotherapy, but for the purposes of this article, the relevant feature is that they both involve talking to a (human) professional. It is in the nature of these therapeutic approaches to mental health that a therapist listens to, understands, and often empathises with a patient.

Machines lack consciousness and emotions, and cannot empathize with us or experience human emotions. So how could AI be of any use in mental healthcare? One way to approach the question is to consider how poorly more traditional ways of approaching mental health have done, compared to other areas of health care. The benefits of AI use in psychiatry need to be assessed against the performance of human therapists and pharmaceutical interventions. If the bar they set is relatively low, then meeting the challenge for AI might be easier than one might think.

Under the wide umbrella of “mental health” issues there are a variety of very different conditions, encompassing anything from mild anxiety disorder to bipolar disorder, from mild depression to schizophrenia. It is not possible to tell if AI could be equally useful in treating all of the different conditions, or only some of them, but it is likely that AI could be at least somewhat useful in addressing the increasing need for mental healthcare worldwide. Indeed, it seems that despite the progress in making healthcare more adequate, individualized, patient-centered, accessible and effective—mental health is not improving both at the global and, in many cases, at the local level. At a global level, poor mental health is estimated to cost $2.5 trillion per year comprising costs of treating poor health and productivity losses. On some estimates, the cost is expected to rise to $6 trillion by 2030 (see Lancet editorial 2020).

In 2022, the *Lancet Psychiatry* published an analysis (GBD 2022) of longitudinal data from 1990 to 2019, comprising 204 countries and looking at 12 mental disorders (various authors 2019). According to this study, there was a 48% increase in diagnoses of mental disorders over the last two decades (from an estimated 654.8 million in 1990 to 970.1 million cases in 2019). Although both males and females suffer from mental disorders in equal measure, some mental disorders affect females more than males (such as depressive disorders, anxiety disorders, and eating disorders), whereas some others affect males more than females (ADHD and autism). Among both sexes, the most common conditions were anxiety and depression. The COVID-19 pandemic restrictions negatively affected mental healthcare at a global level (by depriving people of social interactions, causing some to lose their source of income, etc.) (Gao et al. 2022, Hansen and Menkens 2021). Still, the situation was quite dramatic even before the pandemic. Moreover, inequalities persist. Black people and people from low-income households are less likely to access mental healthcare services in England (McManus et al. 2016) and the US (Hodgkinson et al. 2017).

In sum, despite all the efforts made so far to achieve better outputs for patients, little progress has been made, and indeed, it seems that things have gotten worse.

## How AI can Help to Improve Mental Healthcare

Given concerns about the worsening of mental health at a global level, and the implementation of AI technology in many other areas of healthcare, it is not surprising that attempts have been made to use AI to address mental illness.

At the moment, AI has proved helpful in diagnosing different kinds of mental illness, often via means unavailable to human therapists. For instance, AI can access relevant information about a patient from various sources (medical records, social media posts, internet searches, wearable devices, etc.), and it can quickly analyse and combine the different datasets it has gathered. By identifying relevant patterns in the data, it can help diagnosing mental illness (Walsh et al. 2017). In particular, AI has been used to help with mental healthcare in three main ways^3^, namely (1) through “personal sensing” (or “digital phenotyping”), (2) through natural language processing, and (3) through chatbots (D’Alfonso 2020).

Personal sensing (or digital phenotyping) is the use of digital data to measure and monitor someone’s mental health. For instance, AI can use the material posted on social media, medical records, and so on. By analysing this information, AI can detect relevant behavioural changes that it has learnt to associate with mental health issues. If one wears a smartwatch to track their physical activity and suddenly goes from being very active to being very sedentary, AI technology might take this as a symptom of depression (depressed people often feel lethargic and unmotivated to exercise (Brunell 1990)).

Natural language processing algorithms track the use of language in conversations (chats, emails, social media posts) and detect patterns that might correlate with mental issues such as depression or anxiety. They can also be used to detect changes in the language and to track a patient’s mental health, to see if they are improving or regressing. The very widespread use of smartphones makes natural language processing a relatively cheap way to track mental health^4^. Most people have smartphones and regularly use them to communicate with friends and family, read the news, make online purchases, take pictures and sometimes even work. They therefore contain a significant amount of personal data, which makes them a convenient and practical tool for detecting linguistic patterns that can be linked to certain mental conditions.

Besides natural language tracking, there are claims that it is also possible to detect depression using the patterns in smartphone typing, without even relying on the content being typed (Mastoras 2019, Narziev et al. 2020). This is because, allegedly, depression impacts how we move our bodies, including how we type, so machine learning can be used to detect and identify specific patterns linked to depression or other conditions (it has been noticed, for example, that longer intervals between calls or messages, as well as shorter phone calls, can be a warning sign of relapse of schizophrenia (Buck et al. 2019)).

Finally, some studies suggest that chatbots are able to detect mental issues by asking questions in the same way a mental practitioner would (Vaidyam et al. 2019). The chatbot might ask questions about someone’s mood, stress levels, energy levels, sleep patterns, and so on (Denecke et al. 2021). The chatbot can analyse the patients’ answers and suggest different kinds of therapies (including purely behavioural changes, such as walking, meditating and relaxation techniques) or propose to seek medical advice (if pharmaceutical intervention is considered the most adequate kind of treatment). In some cases, if there are concerns for the immediate safety of the patient or those close to her, the chatbot could send an alert to the patient’s medical practitioner. Something similar is done already with smart glucose trackers: if the glucose monitors senses that glucose is too high or too low, it sends an alert to the medical team, so that they can get in touch with the patient.

In sum, there seems to be at least some potential for AI as a beneficial tool in the provision of mental healthcare. The cost in terms of dehumanisation healthcare delivery might be worth paying, after all, *if* AI can live up to the expectations that have been set. This is a big “if’, and we are happy to leave the question open as to whether AI can deliver what the studies reviewed above seem to promise. Even if someone is skeptical, however, it is likely that AI *will* be employed more widely, given the current trend in the implementation of AI in many professional areas, including healthcare. Yet at this stage, it is hard to predict exactly how new technological advancements will change the way mental illness is detected, diagnosed and treated through the use of AI.

It is also not possible to tell whether AI will improve mental healthcare provision in all countries, or only in those countries where resources are particularly scarce, or where stigma around mental illness is very widespread. Here, we have been concerned primarily with the possible use of AI to solve some long-standing problems in mental healthcare provision. In the following paragraphs, we will review the benefits and drawbacks of using AI, as well as some of the ethical and philosophical implications for mental healthcare delivery.

## Lack of Self-Awareness

One of the reasons people don’t seek help when they suffer from mental health issues is that they are often unaware of their changed mental health status (Gilleen et al. 2010). For instance, some common symptoms of depression, such as fatigue, headaches and back pain, are not immediately linked to mental illness, and they might be misinterpreted as simply the result of lack of sleep, over-exercising, or unhealthy diet. In such situations, one might not seek medical advice and try to self-medicate with off-the-counter medications. These might fix the physical symptoms, at least temporarily, without addressing the root cause. Obviously, healthcare practitioners can’t do much for someone who doesn’t seek help. However, AI-based tools could help make people more aware of their mental health status and keen on seeking professional help.

We saw above how apps keeping track of typing patterns on one’s phone, or the frequency and length of phone calls, are believed to be able to detect the inception of some mental illnesses well before a patient might notice that they need help. Such apps can send a message to the user, advising them to seek medical help because they might suffer from a certain condition. A healthcare professional might then assess whether the patient actually needs medical treatment or whether the app misinterpreted some behavioural patterns. An app monitoring online behavioural patterns could therefore ensure that certain conditions are detected early on and, in some cases, that they are detected at all, such as when patients lack sufficient self-awareness to seek medical intervention.

One obvious downside of digital phenotyping is that there would probably be a lot of false positives. This, in turn, could unnecessarily burden health systems, increasing costs and inefficiencies. Yet the problem could be avoided if the technology becomes advanced enough to avoid false positives. However, the relevant question to ask in order to assess AI performance in this case would not be ‘how many false positives does it produce?’. Rather, it would be ‘do the costs of such false positives outweigh the costs of both false positives and false negatives that human therapists produce?’. As we noted above, the bar might be set low enough to make even a mediocre AI good enough to improve mental healthcare delivery and outcomes. Issues related to over-diagnosing could be solved by developing better algorithms, but it is not possible to say at this stage how accurate such algorithms will prove to be.

It is also possible that promoting a conversation about mental health at the societal level will increase mental health awareness, making AI useless in this respect. But at the moment, this lack of self-awareness can be an obstacle to looking for a treatment, suggesting a role for AI (Metz 2018).

## Social Stigma

In some cases, people are aware of their mental illness and can afford the psychological support they need, but don’t ask for help because of the stigma around mental illness (Corrigan and Watson 2002). In general, society tends to be more supportive of people suffering from physical health issues rather than psychological ones, even though they can both cause severe suffering (Noordgren, Banas and MacDonald 2011).

AI could help alleviate this problem by providing help without any need for the patient to disclose their issue to another human being. Virtual mental health therapists or chatbots can provide mental health support, and they can also provide diagnoses and recommend therapies. It’s possible that patients worried about social stigma would feel more comfortable asking an AI for help rather than a GP or a human psychotherapist. For patients who are seriously concerned about being stigmatized because of their mental illness, the alternative might be between being cured by an AI and not being cured at all.

However, AI would be of no use to someone who has internalised such stigma to the point that they refuse to interact even with an AI, or reject the diagnosis the AI might give, and/or refuse treatment. It’s only in those cases where patients are willing to accept that they may suffer from mental issues and need treatment that AI could be helpful. But this could turn out to be a large enough number of people to make the development of AI in healthcare worthwhile.

## Preference for Avoiding Human to Human Interaction

Some conditions, such as depression or autism, can make interactions with other humans quite challenging. People suffering from depression can sometimes find it difficult to leave the house to get medical assessment, or to go see a therapist.

People with autism can find interaction with other humans very difficult, especially with people they don’t know. In patients that struggle with human interaction, AI could be a more useful tool than a psychotherapy session with a human doctor. According to the studies mentioned above, the AI could diagnose the condition through an app or a chatbot, and could also offer support through a computer. For instance, children with autism could use videos generated by the AI in order to acquire certain competences, and then test such competences in the real world once they feel confident and ready.

Researchers also found that soldiers are more likely to open up about post-traumatic stress when interviewed by a virtual interviewer, and that virtual interviewers were better than human ones at obtaining more medically relevant information from veterans (Fiske, Henningsen and Buyx 2019). Indeed, some research suggests that such virtual therapists are relatively successful in alleviating the symptoms of post-traumatic stress disorder (Lucas et al. 2017). And robot therapists made patients more open to engage in talk therapy (Fiske Henningsen and Buyx 2019). We can’t assume that this is the case for all patients, of course. In most cases, the connection with another human is an essential part of the treatment. However, in those cases where the presence of another human can hinder recovery, it is worth considering the use of AI.

## Lack of Resources

The number of people suffering from mental issues keeps increasing every year, including among children^5^. Yet the number of healthcare practitioners available worldwide cannot grow at the same speed, and is already insufficient to cover everyone’s needs. Especially in middle- and low-income countries, the number of healthcare practitioners is considerably below the needs of the population (Gureje and and Lasebikan 2006; Essien and Asamoah 2020). In Western countries the situation is better, though far from ideal. According to the World Health Organization, there is a global shortage of 4.3 million mental health workers, and it is estimated that the shortage will reach 10 million by 2030 in low- and lower-middle-income countries^6^. Healthcare practitioners seem to be aware of this situation, and perhaps this is why one survey found that, across 22 countries, the majority of psychiatrists perceived AI as a possible solution to the shortage of personnel (Doraiswamy et al. 2020).

A shortage of healthcare practitioners is likely to have a negative impact on the treatment of mental illnesses, because many people in need of medical support will not be able to get the assistance they need. As we saw already, AI could help diagnose and treat patients through apps that are easily installed on a smartphone or through chatbots that can aid psychotherapy. AI robot might be programmed to interact with a patient in a way that resembles the interaction of a human psychotherapist. If such an AI existed, it would be able to ask questions (using an electronic voice) and to understand the patient’s answers, and would also be able to make the conversation progress towards achieving certain results. This would seem to go a great length at making up for the shortage of therapists.

Since AI would be made available to anyone who owns a smartphone and can access the internet, a minimum standard of care could be guaranteed to a much wider portion of the population—while those willing to pay for human therapists would still have the option to do so.

## Inefficiency

One of the main downsides of “traditional” mental healthcare is its relative inefficiency. Even when a diagnosis is available, treatments are not always adequate to cure a certain mental illness. For instance, a recent study suggesting that anti-depressants are only marginally more effective than placebo raised a lot of attention (Almohammed et al. 2022) .Often patients have to try different anti-depressants before they find one that works for them without causing side effects that outweigh the benefits (Le Pen et al. 1994). In some cases, the patient might give up before the best treatment is found, so they end up not using any treatment at all, thereby worsening their condition (Demyttenaere et al. 2001).

It’s not yet clear why some anti-depressants work better on some individuals rather than on others. One hypothesis is that genetic differences might make some people more responsive to certain drugs (Tansey et al. 2013). It has been suggested that AI could be employed to gather information about the genetic characteristics of individuals that respond better to antidepressant X or Y, and then match the genetic information of the patient to the most effective therapy (Drysdale et al. 2017). Not only could the AI identify the best pharmaceutical treatment for a given patient, but it could also suggest the most appropriate non-pharmaceutical treatments. For instance, if the behavioural or genetic profile of the patient, or their symptoms, suggests that the patient won’t respond well to a pharmaceutical approach, the AI could suggest deep brain stimulation or cognitive therapy. Indeed, machine learning can already predict effectiveness of deep-brain stimulation in the treatment of different types of mental illness (Drysdale et al. 2017).

Lack of efficacy of antidepressants is not the only reason to take seriously the potential benefits of AI. There are some kinds of mental illness that are notoriously hard to treat even for experts. It is well-known that psychiatrists find it particularly difficult to assess whether a patient is likely to attempt suicide. According to a meta-analysis covering 365 studies published over the past 50 years, psychiatrists are only marginally better than chance at predicting suicide (Franklin et al. 2017). Researchers have developed an algorithm that they claim can predict whether someone will attempt suicide within the next 24 months, and can do so with accuracy around 85%. Within the timespan of a week, it can predict a suicide attempt with 92% accuracy (Walsh et al. 2017). This result was obtained through the use of large datasets, the analysis of medical records, and the tracking of social media posts. It would therefore seem that when it comes to predicting suicide, empathy and experiencing human emotions in general may be less important than having access to large amounts of small bits of information that AI could gather and process better than humans (Loh 2018).

The shortcomings of treatments based on antidepressants, as well as the difficulties encountered by doctors in anticipating suicidal attempts by patients should not lead us to conclude that current mental healthcare is ineffective in absolute terms. Rather, identifying the difficulties of mental healthcare practitioners can help understand when and how to use AI effectively. Identifying the best anti-depressant using genetic data, and predicting whether a suicide attempt will occur, are tasks where AI seems to perform better than humans at the present time. In cases where AI can enhance the efficacy of healthcare, there are more compelling reasons to use it than in cases where AI is equivalent to, or less effective, than human counterparts.

## Healthcare Practitioners’ Bias

Treating all patients with impartiality and objectivity, are goals of all healthcare practitioners. Yet we humans, including healthcare practitioners, are prone to partiality and bias, and this can sometimes affect the quality of the healthcare provided (FitzGerald and Hurst 2017). For instance, it has been reported that autism in women is under-diagnosed, possibly because, being less common among women, practitioners tend to assume that relevant symptoms in women are not linked to autism (Zener 2019). This, in turn, raises the question of whether or to what extent under-diagnosis can explain why it is believed to be less common in women.

Individual and social factors such as age, social status, ethnic background or past medical history can mislead the practitioner when performing a diagnosis. Although taking into account such factors can often increase the accuracy of a diagnosis, it is also possible that attributing them too much importance can hinder the accuracy of the diagnosis. A human practitioner may find it difficult to disregard certain information about the patient and focus exclusively on the symptoms.

AI could be instructed to perform different types of diagnosis, for instance, one based exclusively on symptoms, and another one that takes into account sex, age, etc. as well as genetic factors or information collected through the use of wearables. Such diagnoses could be matched and compared with that of a human healthcare practitioner, potentially leading to a more accurate diagnosis for the patient, and hence a quicker recovery.

However, one must remember that AI, being programmed by humans, can be biased itself (Parikh et al. 2019), and it is important to be aware of the potential biases in the algorithm and to adjust its analysis accordingly.

## Mental Health Categories and Responsibility for Diagnoses

All of the issues discussed so far have been widely debated over the past few years. Recent developments in AI could raise concerns and doubts on one hand, but also make us hopeful about the possibility of making mental healthcare more effective and widely available. Here we want to discuss two ethical-philosophical considerations that need to be given some thought before AI is implemented (Giubilini 2021), on the assumption that AI delivers on what it seems to be promising—an assumption we do not challenge here, but which does call for healthy skepticism.

The first consideration is about the way we categorize mental health disorders. The Diagnostic and Statistical Manual of Mental, currently in its 5th edition (DSM5), is the most widely used diagnostic tool. The symptomatology and behavioural cues on which it relies to categorize mental disorders are based on human capacities for detection and assessment. Such capacities can be fine-grained up to a point. But if AI is able to deliver more fine-grained information and thus live up to expectations, for example by including speech patterns among the behavioural cues or symptoms, it is not clear that DSM categories will still be fit for purpose. Conversely, one might conclude that the AI is not fit for purpose, if we think our current categories of mental health disorders are worth preserving. Whichever approach is taken, we need to reflect on the usefulness of current mental health categories in light of ongoing developments. In this sense, the introduction of AI in psychiatry might bring a new perspective on the already wide literature criticizing the DSM5’s approach to categorization of mental health disorders (Hyman 2010).

The second consideration is the new responsibilities that the use of AI specifically in mental health care, as opposed to healthcare more generally, confers on mental health professionals. Perhaps the most widely discussed issue in the literature on AI in healthcare, alongside that of the potential biases of algorithms, is that of potential gaps in responsibility for mistakes (Mishra et al. 2021; Kiener 2022; Grote and Berens 2020): if an algorithm makes a mistake, do human therapists have a responsibility for rectifying it and, if not, who really *is* responsible? We will not address this problem in detail here. Rather, we want to point to a somewhat separate issue about responsibility that arises specifically in the mental healthcare context.

In most cases, AI in healthcare is concerned with well-defined disease categories, such as oncology or cardiology. Mistakes can occur when clear and largely unquestioned categories are applied to the wrong set of circumstances. Yet as we noted, AI might raise questions about the suitability and meaningfulness of such categories themselves. There is a substantial difference between incorrectly applying a valid standard (or at least one widely acknowledged to be valid by the relevant expert community, as is the case with oncology categories) and making decisions using a standard that might not be (acknowledged as) valid. If AI creates a situation where we can no longer rely on valid standards for diagnoses because the old standards are no longer fit for purpose, what would it even mean to misdiagnose a mental health condition, and on what basis could a practitioner be held responsible? AI introduces a level of uncertainty around standards of correct diagnoses that might require redefining the scope of responsibility in mental healthcare. Practitioners might be required to modify or reinvent categories of mental disorders as they operate with new types of data provided by AI. This might imply they have the responsibility not only for correct diagnoses, but for correctly modifying the criteria of correct diagnoses as they operate. However, it is not clear at this stage how such criteria could be modified in light of the new types of clinically relevant information, since professionals are not used to thinking of things like speed of typing or most social media content as clinically relevant. And it is not clear that the task of redefining such categories falls within practitioners’ professional obligations.

## Conclusion: Should We Use AI in Psychiatry?

It is not possible to answer the question about whether and to what extent AI should be adopted in mental healthcare. Too much information is missing about both its potential benefits and its potential drawbacks. However, it would make sense to use AI to support mental healthcare provision if and when there are good reasons to think AI outperforms or can significantly assist human therapists. These are largely empirical issues which we are not going to address. But we will provide some general guidelines that could prove useful once more information is available.

There are several possible scenarios that could play out:

1. It might turn out that using AI greatly improves outcomes for patients, with relatively small downsides. This is the most optimistic scenario, as it would solve a sizeable share of problems by providing a large number of people with better mental healthcare at sustainable costs.
2. It could turn out that AI greatly improves healthcare provision, but with downsides so significant that it wouldn’t be worth using. In this scenario, AI would be useful, but it would be too expensive, or would require excessive use of personal data, or would entail excessive deresponsibilization of practitioners, etc. In such a scenario, the benefits would outweigh the costs only in those cases where humans perform *significantly* worse than AI, but such cases mightbe difficult to identify.
3. It might turn out that the best results can be obtained through the collaboration of AI and healthcare practitioners. AI might never develop human emotions that could allow it to fully understand the emotions of a patient. Human practitioners might never be able to cover the growing demand for mental healthcare at the global level. A recent study asked experts to compare the answers provided by ChatGPT and healthcare practitioners to some medical questions posted online to various forums (Ayers et al. 2023). The researchers found out that the answers provided by ChatGPT were rated higher for both quality and empathy by the experts, whereas the answers by the physicians were shorter and less detailed. The authors concluded that it would be useful to explore the use of ChatGPT in clinical settings, for instance allowing the AI chatbot to draft answers that are then checked and edited by the doctors. We can easily imagine how the health system could become more efficient if some of the burdens were removed from the human practitioners and shifted to the AI (for instance, the initial anamnesis of the patient’s medical history could be conducted by the AI, and the results analysed by the healthcare practitioner).
4. It might turn out that, despite initial positive outcomes, AI is not as cost-effective as we thought; and that humans are actually better than AI at performing diagnoses and administering treatments. It might be the case that innovation and improvement in healthcare aren’t really possible unless humans are involved. A certain amount of trial and error has been necessary to get to the point where we are now, but if medical practice were relinquished to machines, we might find ourselves with a lack of new development and stunted progress overall. We might also find out that human relationships are an essential part of healthcare, and that outcomes are worse if patients are not given the option of interacting with other humans. We might also conclude that allowing AI to access sensitive data about one’s health poses risks that we can’t fully understand and foresee. In this scenario, we would need to rethink whether to use AI in mental health care at all.

## Footnotes

1
https://ts2.space/en/chatgpt-in-the-entertainment-industry-revolutionizing-scriptwriting-and-storytelling/.

2
https://www.bbc.com/news/health-50857759.

3
https://www.sciencedirect.com/science/article/pii/S2352250X2030049X.

4
https://www.psychologytoday.com/intl/blog/different-kind-therapy/202211/ai-can-use-your-voice-detect-depression#:~:text=AI%20can%20be%20a%20useful,concerns%20about%20privacy%20and%20ethics.

5
https://www.theguardian.com/society/2023/jan/03/child-referrals-for-mental-health-care-in-england-up-39-in-a-year.

6
https://www.who.int/health-topics/health-workforce#tab=tab_1.
